# Medicine

**Published:** 1898-12

**Authors:** J. Michell Clarke


### Progress of the Medical Sciences.

### MEDICINE.

In the discussion on treatment of **diseases of the stomach** at the Edinburgh meeting of the British Medical Association several of the speakers alluded to the **importance of rest.** In that form of dyspepsia which occurs in the overworked, Dr. Wm. Calwell[1] emphasised the necessity of absolute rest of body and mind in bed for a period of from two to ten days, together with a carefully regulated diet, beginning with a small teacupful of cooked diluted milk every two hours, gradually increasing to a fairly full diet; and some mild alkaline sedative before food. Prof. Saundby said that he thought that neither the profession nor the public quite appreciated the value of rest in the treatment of diseases of the stomach. Treatment by the rest cure, however, as Prof. Ewald pointed out, whilst bringing about a considerable gain in weight, due to retention of water and deposit of fat, does not always relieve the dyspeptic symptoms. Such patients require a thorough course of roborant treatment in the mountains or on the sea-coast, or in a well-situated sanatorium for dietetic and hydrotherapeutic treatment. Dr. Spivak[2] advocates absolute rest with a suitable dietary in all dyspepsias the underlying cause of which is a deranged action of the nervous system; in all cases where abdominal pain is present; in all cases of acute and chronic diarrhœa; in hemorrhage from the gastro-intestinal tract; in all tubercular cases suffering from disturbed digestion.

In **motor insufficiency (atony) of the stomach** Boas[3] gives the following hints for dietetic treatment, classifying the cases thus: (1) Insufficiency with permeable pylorus and retained (increased) secretion of gastric juice, needs a restriction of the fluids allowed; (2) contracted pylorus, normal (increased) secretion of gastric juice, indicates that fluid and soft food will be

[1] *Brit. M. J.*, 1898, ii. 1330. [2] *J. Am. M. Ass.*, 1898, xxxi. 216.
[3] Quoted in *Ibid.*, 1897, xxix. 442.

better digested, especially milk in small quantities; (3 and 4) suppression of the gastric juice, carcinoma and severe gastritis. Fluid food is best in all cases, and milk should be always tried. In benign stenosis with abundant gastric juice, the best diet is animal albumin with restricted non-nitrogenous food; in stenosis of the pylorus, with lack of gastric juice, carbohydrates and fats are preferable, with easily assimilable vegetable albumin. Feeding *per rectum* must be carried out if it is impossible to maintain the nutrition by the food taken into the stomach. As the tissues dry out rapidly in such cases, the introduction of salt solution is valuable, also of alimentary fluids.

With regard to the administration of **peptonised foods,** Dr. Herschell[1] stated that "according to present ideas, it is quite superfluous to give peptonised albumins as long as the motor power of the stomach is normal, and as long as we are certain that duodenal digestion is unimpaired. Even when the power of the stomach of emptying itself into the duodenum has been lost, as in cases of pyloric stenosis, we shall probably be wasting our time in converting the food into peptone since the investigations of Cahn have demonstrated that in these cases no peptone is absorbed from the stomach." The use of peptone and albumose is to be confined to cases where the ordinary diet is found to be insufficient to preserve the nitrogenous equilibrium of the body.

In cases of **hyperchlorhydria**, hypersecretion of the gastric juice—a condition in which the stomach secretes an excessive amount of gastric juice at a time when digestion is not in progress, especially during the night, this gastric juice being either normal or containing an excessive amount of HCl,—Prof. Ewald[2] first employs tonic and hygienic regulations for the general condition, and, secondly, regular evacuation of the contents of the fasting stomach, and the introduction by means of Einhorn's stomach spray or the stomach douche of a half per cent. solution of $AgNO_3$. During the day he gives the patient every two hours a teaspoonful of a five per cent. solution of potassium iodide and bicarbonate of soda, and allows only rectal alimentation, in order to avoid all irritation of the gastric mucous membrane. He has found, after a large experience, the ordinary sedatives, bromides, zinc, belladonna, codeia, morphia, and hyoscyamus, of no use. Dr. Joslin[3] has found tincture of nux vomica the only drug of value in this condition. He gives ten drops three times a day, increasing one drop daily until a maximum daily dose of sixty to ninety drops is reached. At the same time outdoor physical exercise is enjoined and massage employed where the motility of the stomach is at fault. Should these measures fail, the stomach tube should be used.

Recent work has demonstrated that patients may remain healthy and maintain their nutrition in the presence of great

[1] *Brit. M. J.*, 1898, ii. 1322. [2] *Ibid.*, 1324.
[3] *Boston M. & S. J.*, 1898, cxxxviii. 389.

**deficiency of the gastric secretion**, so long as the motor power of the stomach is sufficient to promptly carry on its contents into the intestine. If, however, the muscular power of the organ fails, dilatation is gradually established and increases in amount, and all the consequences of retention and abnormal fermentation of the stomach contents follow. According to Prof. Ewald, the only internal remedy for failure of motor power is strychnine. Something can be done for stagnation of food in the stomach by lavage, stomach douches, massage vibration and electricity. He warns against the too frequent employment and too long continuance of washing out the stomach: where it does good, it does so soon.

With regard to the value of **electricity** there is considerable difference of opinion. Dr. Herschell treats cases of atony of the stomach mainly by the application of the continuous current to the solar plexus and to the ganglia of the sympathetic and vagi in the neck. He also advocates the use of a primary coil with slow interruptions of the current. Ducceschi found,[1] from experiments on dogs, that the influence of electrical stimulation on the stomach was in close relation with the functional condition of its muscular wall. Strong contractions were excited in the condition of rest, they were weaker or failed altogether in that of activity. Dr. Boardman Reed[2] says that a strong faradic current applied by means of an intra-gastric electrode is very efficient in strengthening the gastric muscle and contracting the dilated organ. There resulted, however, in all except one of his cases during this treatment a depression of the secretion of HCl. The treatment did best in cases of dilatation with chronic gastric catarrh and very deficient secretion.

In the milder cases of gastrectasia or, as he prefers to call it, **ischochymia** Dr. M. Einhorn[3] employs a fluid or semi-fluid diet (milk, soups with finely ground farina, meat broths with egg, egg and milk), lavage of the stomach in the fasting state, followed by spraying the stomach with $\frac{1}{1000}$ solution of $AgNO_3$, and the administration of medicines to check fermentation, especially the following mixture: Resorcin 4, Bismuth. subnit. 20, Aq. destill. 200; ℥ss three times a day before meals. In severe cases it is advisable to keep the patients in bed for three weeks, and to nourish them exclusively *per rectum* for five days, and then slowly and cautiously adopt a milk diet, beginning with two tablespoonsful of milk every hour, three tablespoonsful on the next day, and so on until 100 c.c. milk are reached: then 200-300 c.c. are given every two hours. The stomach is washed out every other day. Ischochymia, produced by malignant disease or a benign markedly developed narrowing of the pylorus, demands operative treatment.

1 *Gazz. d. Osp.*, 1896, xvii. 1249; abstract in *Centralbl. f. innere Med.*, 1898, xix. 18.

2 *J. Am. M. Ass.*, 1898, xxxi. 220.

3 *Ibid.*, 1897, xxix. 266.

Dr. Turck[1] uses an instrument which he named a "gyromele," which is a revolving sound for **exploring the stomach,** removing adherent material from its walls, and for locating strictures from the œsophagus to the cardia. It consists of a flexible cable, to the end of which is attached a sponge covering a spiral spring, which can be removed and changed. The cable passes through a rubber tube, and this again is attached to a revolving apparatus for the purpose of producing revolutions of the sponge within the stomach. The advantages claimed are: that the massage of the revolving sound produces an effect upon the vascular supply of the stomach wall; that the mucous membrane can be cleaned of adherent mucus, decomposing remnants of food, and epithelium, which can be then washed out; that various medicaments can be directly applied by it; and that it can be used as a very convenient electrode, capable of application to any part of the stomach it is desired to reach. Dr. Herschell[2] has seen much benefit in cases of atony of the stomach from alternate douching with hot and cold water. Double tubes are used for this purpose, such as those devised by Turck and by Hemmeter.[3]

The following procedure is adopted by M. Albert Robin[4] in cases of **pyloric stenosis** with extreme cachexia in which the diagnosis between a malignant stricture and a spasmodic stricture (due to ulcer, hyperacidity of gastric juice, etc.) is uncertain. He puts the patient on an absolute milk diet, giving 300 grammes of sterilised milk during the day in small quantities every two hours; together with the most complete rest. Given in this way, all patients can take milk well. For the first three or four days the patient loses weight, the following days he regains it, and on the eighth or ninth day he ought to gain weight progressively. If, on the other hand, the patient's condition remains stationary or deteriorates, surgical procedures should be adopted. In cases of great dilatation, a preliminary washing out of the stomach is necessary. Although advising lavage in such cases, he cautions against its too prolonged or too frequent employment.

About the efficacy of **massage** in the treatment of motor insufficiency of the stomach there is a difference of opinion. According to Hemmeter,[5] it may be used on an empty stomach before breakfast to strengthen the muscular power of the stomach, or three or four hours after a meal to assist in the expulsion of its contents; and may be usefully employed in simple atony, pyloric stenosis of moderate degree, chronic gastritis, gastroptosis, and in certain cases of nervous inhibition of the peristaltic movements.

J. MICHELL CLARKE.

[1] *Wien. med. Wchnschr.*, 1895, xlv. 8, 64; abstract in Sajous's *Annual*, 1896, i. C–19.

[2] *Loc. cit.* [3] For a description of such tubes, see *N. York M. J.*, 1895, lxi. 385.

[4] Quoted by MM. Coyon et Legros, "Sténoses du pylore," *Gaz. d. Hôp.*, 1898, lxxi. 947.

[5] Quoted by Dr. Herschell, *loc. cit.*

